# Intra-Myocardial Injection of Both Growth Factors and Heart Derived Sca-1^+^/CD31^−^ Cells Attenuates Post-MI LV Remodeling More Than Does Cell Transplantation Alone: Neither Intervention Enhances Functionally Significant Cardiomyocyte Regeneration

**DOI:** 10.1371/journal.pone.0095247

**Published:** 2014-06-11

**Authors:** Xiaohong Wang, Qinglu Li, Qingsong Hu, Piradeep Suntharalingam, Arthur H. L. From, Jianyi Zhang

**Affiliations:** Cardiovascular Division, Department of Medicine, University of Minnesota Medical School, Minneapolis, Minnesota, United States of America; Georgia Regents University, United States of America

## Abstract

Insulin-like growth factor 1 (IGF-1) and hepatocyte growth factor (HGF) are two potent cell survival and regenerative factors in response to myocardial injury (MI). We hypothesized that simultaneous delivery of IGF+HGF combined with Sca-1^+^/CD31^−^ cells would improve the outcome of transplantation therapy in response to the altered hostile microenvironment post MI. One million adenovirus nuclear LacZ-labeled Sca-1^+^/CD31^−^ cells were injected into the peri-infarction area after left anterior descending coronary artery (LAD) ligation in mice. Recombinant mouse IGF-1+HGF was added to the cell suspension prior to the injection. The left ventricular (LV) function was assessed by echocardiography 4 weeks after the transplantation. The cell engraftment, differentiation and cardiomyocyte regeneration were evaluated by histological analysis. Sca-1^+^/CD31^−^ cells formed viable grafts and improved LV ejection fraction (EF) (Control, 54.5+/−2.4; MI, 17.6+/−3.1; Cell, 28.2+/−4.2, n = 9, P<0.01). IGF+HGF significantly enhanced the benefits of cell transplantation as evidenced by increased EF (38.8+/−2.2; n = 9, P<0.01) and attenuated adverse structural remodeling. Furthermore, IGF+HGF supplementation increased the cell engraftment rate, promoted the transplanted cell survival, enhanced angiogenesis, and minimally stimulated endogenous cardiomyocyte regeneration in vivo. The in vitro experiments showed that IGF+HGF treatment stimulated Sca-1^+^/CD31^−^ cell proliferation and inhibited serum free medium induced apoptosis. Supperarray profiling of Sca-1^+^/CD31^−^ cells revealed that Sca-1^+^/CD31^−^ cells highly expressed various trophic factor mRNAs and IGF+HGF treatment altered the mRNAs expression patterns of these cells. These data indicate that IGF-1+HGF could serve as an adjuvant to cell transplantation for myocardial repair by stimulating donor cell and endogenous cardiac stem cell survival, regeneration and promoting angiogenesis.

## Introduction

The left ventricular (LV) remodeling that occurs following myocardial infarction (MI) results, in part, from the abnormal LV wall stresses that develop in surviving myocardium. The increased wall stress is thought to induce adverse molecular responses in the residual myocardium [1]–[3]. Importantly, the limited ability of the heart to regenerate lost cardiomyocytes and vascular cells contributes to the severity of LV remodeling. Therefore, administration of various types of presumed cardiac regenerative cells including skeletal muscle myoblasts, marrow derived mesenchymal stem cells (MSCs), endogenous cardiac stem cells (CSCs), endothelial progenitor cells, induced pluripotent stem cells (iPSCs) and embryonic stem cells to hearts following acute infarction (acute MI) has been attempted in the hope of stimulating cardiac regeneration [4]–[9]. It is well known that many animal and clinical trials have indicated that cell transplantation modestly improves cardiac function in post-MI hearts. However, in most animal studies persistent engraftment of transplanted cells has been minimal and few of the transplanted cells appear to have proliferated and differentiated into new cardiomyocytes or vascular cells [10]–[12].

The microenvironment in acutely injured myocardium has been considered to be hostile to both donor cell and native CSCs survival and propagation because of the presence of hypoxia, acidosis, inflammatory mediators, and reactive oxygen and nitrogen species [13]–[14]. Hence, attempts to ameliorate this “transplantation hostile” state have been made including the injection of insulin-like growth factor I (IGF-I) and hepatocyte growth factor (HGF) into the acutely injured heart. IGF-1 and HGF are potent cell survival and regeneration factors [15]–[16] and cardiac restricted over-expression of IGF-1 increases the formation of ventricular myocytes and attenuates myocyte death [17]–[18]. IGF-1 receptor activation induces division of CSCs, upregulates telomerase activity, and preserves the pool of functionally competent CSCs [17]–[18]. HGF also enhances survival of mature cardiomyocytes under ischemic conditions [19]–[20]. Moreover, intramyocardial HGF gene therapy post-MI is associated with increased angiogenesis and preservation of cardiac contractile function [21]–[22].

Consistent with these observations, studies from the laboratories of Anversa and his colleagues have shown, in both large and small animal studies, that the intra-myocardial combined injection of HGF and IGF facilitated survival of endogenous c-kit^+^ CSCs (the majority of which were Sca-1^+^) and enhanced their migration to injured areas, their proliferation, and differentiation into cardiomyocytes and vascular cells [6], [23]–[25]. Significant cardiomyocyte regeneration from CSCs associated with partial repopulation of the infarct scar zone was also present and it is likely that survival of native cardiomyocytes was also increased in these post-MI hearts. In consequence, substantial attenuation of the structural and functional consequences of LV remodeling was present in these studies.

It is well known that Sca-1 is an antigen located in the plasma membrane of many lines of progenitor cells. Hence, we previously studied the effects of intra-myocardial injection of allogenic heart derived Sca-1^+^/CD31^−^ cells into the peri-infarct region of acutely infarcted mouse hearts; we observed attenuation of LV remodeling with the result that cardiac contractile function and energy metabolism were partially preserved [26]. However, evidence of significant myocardial regeneration from engrafted cells was not present in these hearts despite the fact that our cardiac derived Sca-1^+^/CD31^−^ cells were able to differentiate to early cardiomyocytes under *in vitro* conditions. We have also reported that post-MI LV remodeling is significantly worse in the hearts of Sca-1 (heterozygote) knockout mice (Sca-1^−/+^)[27]. Collectively, these data suggested that the presence of Sca-1^+^/CD31^−^ cells in the myocardium improved the response to an ischemic insult.

Based on the aforementioned data, we hypothesized that the simultaneous delivery of IGF+HGF and heart derived Sca-1^+^/CD31^−^ cells into the LV peri-infarction zone after coronary occlusion would attenuate LV remodeling more than would cell transplantation alone and the increased benefits of combined treatment would be due, in part, to increased cardiomyocyte regeneration from transplanted and endogenous CSCs.

We found that IGF+HGF administration combined with cell transplantation significantly increased the rate of Sca-1^+^/CD31^−^ cell engraftment, reduced infarct size, and further attenuated post-acute MI LV structural and functional remodeling as compared to the results achieved by injection of Sca-1^+^/CD31^−^ cells alone. However, in contradiction to hypothesis, there was no evidence that functionally significant numbers of either engrafted Sca-1^+^/CD31^−^ cells or endogenous CSCs differentiated into new cardiomyocytes. Rather, the data suggested that the beneficial effects of transplantation were the result of increased paracrine signaling which resulted in additional protection of native cardiomyocytes from death (apoptotic and/or necrotic) and the maintenance of normal capillary density in the peri-infarct zone of the LV; The latter phenomenon likely helped to preserve the performance of this myocardial region. Data from our *in vitro* experiments support these views because firstly, IGF+HGF treatment enhanced the survival of cultured Sca-1^+^/CD31^−^ cells during serum deprivation induced stress; Secondly, co-culture of atrium derived HL-1 cells with Sca-1^+^/CD31^−^ cells protected the HL-1 cells from death induced by three different stressors (TNFα, hypoxia or H_2_O_2_), and lastly, exposure of Sca-1^+^/CD31^−^ cells to IGF+HGF altered the expressions of a number of trophic factors associated mRNAs; the latter finding might indicate the presence of an increased level of paracrine signaling emanating from these cells.

## Materials and Methods

Wild-type mice (BALB/c, 8–10 weeks) were purchased from Jackson Laboratory (Bar Harbor, ME, http://www.jax.org). The University of Minnesota Animal Care Committee approved all procedures and protocols. The investigation conformed to the Guide for the Care and Use of Laboratory Animals of the Institute of Laboratory Animal Research.

### Sca-1^+^/CD31^−^ and Sca-1^−^/CD31^−^ Cell Isolation and Fluorescence-activated Cell Sorting Analysis

The isolation and expansion of Sca-1^+^/CD31^−^ and Sca-1^−^/CD31^−^ cells were performed with modifications of previously described methods [26], [28]–[29] and these techniques are described in detail in the supporting information (Text S1).

### Determination of the Effects of IGF+HGF on Serum Deprivation Induced Sca-1^+^/CD31^−^ Cell Apoptosis and Proliferation

For determining the effects of IGF+HGF on Sca-1^+^/CD31^−^ cell apoptosis, cells were cultured in cardio-sphere growth medium (CGM) and then placed in 6 well plates. Following an additional 24 hours of culture, groups of these cells were exposed to serum free medium with or the addition of IGF(100 ng/ml)+HGF(100 ng/ml) (R&D Systems, Minneapolis, MN). This treatment was continued for 48 hours and the cells were then stained with Annexin V conjugates (BD Biosciences, San Jose, CA) and assessed by flow cytometry according to manufacture’s directions. For cell proliferation analysis, Sca-1^+^/CD31^−^ cells were synchronized in serum free medium for 12 hours. Group of cells treated with basal medium plus 0.5% FBS for 24 hours served as a control group. The treatment group is the group of cells added IGF(100 ng)+HGF(100 ng) in the basal medium plus 0.5% FBS for 24 hours of culture. Cell proliferation was detected using Cycletest Plus DNA Reagent Kit (BD Biosciences, San Jose, CA) and assessed by flow cytometry. The acquired data was analyzed using commercial software (FlowJo, Ashland, OR) and the mathematical model of Dean-Jett- Fox was used in the cell cycle analysis.

### Superarray Analysis of mRNA Expression Patterns in Sca-1^+^/CD31^−^ Cells Treated with IGF+HGF

For gene array analysis, total mRNA from Sca-1^+^/CD31^−^ cells was isolated using RNeasy columns with mRNAase-free DNAase treatment. 1 µg of total mRNA was reversely transcripted into cDNA using a RT first strand kit following the manufacture’s directions (SABiosciences, Frederick, MD). The PCR array contains a panel of 96 primer sets for a set of 84 relevant genes, plus five housekeeping genes and three mRNA and PCR quality controls. The cDNA template was mixed with ready-to-use RT-qPCR Master. Aliquots of the mixture (25 µl) were introduced into each well of the plate containing pre-dispensed gene specific primer sets. We performed a real-time PCR using a two-step cycling program as follows: 95°C, 10 minutes; 40 cycles for 95°C, 15 seconds and 60°C, 1 minute. The data obtained from RT-PCR array was analyzed using SABiosciences PCR array data analysis software.

### Effects Sca-1^+^/CD31^−^ Cell Co-culture on Stressor (Hypoxia, H_2_O_2_ or TNFα) Induced HL-1 Cell Apoptosis

Sca-1^+^/CD31^−^ cells were plated onto fibronectin-gelatin-coated plates or flasks and cultured in Claycomb medium (Claycomb Laboratory, University of Louisiana) supplemented with 10% fetal bovine serum, 100 U/ml penicillin, 100 µg/ml streptomycin, 0.1 mM norepinephrine, and 2 mM L-glutamine as previously described. Before being co-cultured, HL-1 cells were labeled with Vybrant CFDA SE cell tracer kit (Molecular Probes, Carlsbad, CA). Labeled HL-1 cells were extensively washed and co-cultured with Sca-1^+^/CD31^–^ cells. The cell co-cultures were then incubated for 24 h at 2% oxygen (hypoxia) or 21% oxygen (normoxia) or treated with either 0.05 mmol/L H_2_O_2_, or 40 ng TNF α for 12 hours while being maintained in a normoxic medium. Apoptosis was assessed by staining with Hoechst 33342 (H33342) dye and then quantified by the percentage of apoptotic nuclei (300 cells counted per sample) in the CFDA-labeled subset by identifying cells with H33342 staining.

### Animal Surgery and Cell Transplantation

Adult female BALB/c mice aged 10–12 weeks were employed for this study. They were housed in trios or quartets with food and tap water ad libitum. Myocardial infarction induced by left anterior descending coronary artery (LAD) ligation and cell transplantation is described as in detail in the supporting information (Text S2).

### Echocardiography

Echocardiography was performed 4 weeks after myocardial infarction using an echocardiographic system equipped with a high-resolution ultrasound biomicroscope (Vevo 660, VisualSonics, Toronto, Ontario, Canada) with a 30-MHz transducer [26]. The details are described in the supporting information (Text S3).

### Tissue Preparation for Light Microscopy, H&E, and TUNEL Staining

Mouse hearts that had received LacZ transduced Sca-1^+^/CD31^–^ cells were fixed with 2% paraformaldehyde and subjected to β-galactosidase staining. Serial 8-µm-thick sections of each tissue were cut onto slides. Apoptosis-related DNA fragmentation was determined by the TUNEL assay using the ApopTag Plus Peroxidase *In Situ* Apoptosis Detection Kit (Millipore, Temecula, CA) following the manufacturer's instructions. Slides were counterstained with hematoxylin for morphologic definition, dehydrated in a series of ethanols and xylene, and mounted under a glass coverslip. Specimens were examined and photographed with an Olympus BX51 light microscope. Images were captured with the DPManager imaging software. On TUNEL stained sections, apoptotic cells were enumerated by counting 100 cells in randomly selected infarction fields of a total of 10 sections. One thousand cells were enumerated at each heart and the apoptotic index was expressed as the percentage of apoptotic cells per 100 cells enumerated.

### Cell Engraftment Rates and Differentiation Status

The evaluation of cell engraftment rates and differentiation status are described in detail in the supporting information (Text S4).

### BrdU *In situ* Hybridization

A 10 mg/ml solution of BrdU in sterile 1x DPBS was provided for in vivo use. Intra-peritoneal injection of BrdU [200 µl (2 mg)] was injected per day for 10 days following LAD ligation. Groups of animals were sacrificed on day 3 and day 10 after myocardial infarction, and analyzed for BrdU incorporation. BrdU incorporation was detected in cardiac tissues according to the manufacture’s directions.

### Analysis of Capillary Numbers

Capillaries were counted at a magnification of 20x using an Olympus microscope (Olympus BX51/BX52). Infarct border zones were examined for the number of CD31 antibody stained capillaries. The quality of the computer analysis of capillary numbers (NIH Image J program, http://rsb.info.nih.gov/ij) was checked against manual counting. The capillaries were counted in blinded fashion on 50 sections (two fields per section, five sections per heart, *n* = 6 for each group) in the peri-infarct zone.

### Scar Size Measurements

Four weeks after the cell transplantation, the left ventricle was excised, cut open from base to apex, and laid on a flat white paper. Infarct size was calculated and expressed as a percentage of LV surface area. Color digital images were obtained, and the ratio of LV scar area to total LV surface area was evaluated with an image software analysis system (NIH ImageJ program, http://rsb.info.nih.gov/ij).

### Statistical Analyses

Comparisons among three groups were analyzed using one-way analysis of variance followed by Student-Newman-Keuls post hoc tests (*p*<.05). Data are shown as mean ± standard error.

## Results

### IGF+HGF Co-administration with Sca-1^+^/CD31^−^ Cells Attenuates LV Structural and Functional Remodeling more than does Cell Therapy Alone

**Figure 1:**
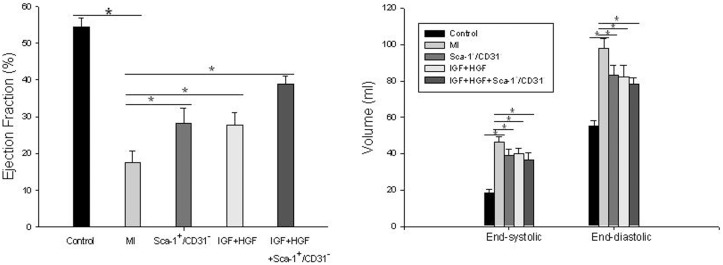
Effects of interventions on LV ejection function and geometry. (**A**)**.** Ejection fraction (n = 9; P<0.01 vs MI). (**B**)**.** LV end-systolic and end-diastolic volume (n = 9, P<0.05 vs MI). Data are presented as mean+/−SEM.

Echocardiography performed four weeks after acute MI revealed significantly higher values of LVEF and better preserved LV geometry in the cell treated group as compared to the control acute MI group (Figure 1) and IGF+HGF + cell therapy further attenuated LV remodeling. LVEF was 19% in the untreated MI group, 28% in the cell treated MI group and 39% in the IGF+HGF plus cell treated MI group (n = 9, P<0.01 vs untreated and cell treated MI groups). Of interest, the dose of both IGF and HGF given to each heart ranged between ∼5 and 7 ng/heart and this dosage was comparable to that employed in several reports that showed significant cardiac regeneration from endogenous CSCs following growth factor administration [17]–[18]. We also found that administration of IGF+HGF without cells was as effective as was cell transplantation alone. However, combination therapy was more effective than either individual intervention. Significant effects were absent in a group transplanted with Sca-1^−^/CD31^−^ cells (data not show), although the values in these groups did trend higher as compared to untreated post-acute MI hearts (Figure 1). Increased wall thicknesses were present in non-infarcted regions of treated hearts (Figure 1) and scar sizes were also smaller in the treated MI hearts (MI, 35.7+/−0.02%; MI+IGF+HGF, 27.12+/−0.09%; MI+Sca-1^+^/CD31^−^cells, 24.9%+/−0.02%; MI+IGF+HGF+Sca-1^+^/CD31^−^cells, 19.9%+/−0.02%; n = 6, p<0.05 vs all other MI groups). It should be noted that no intervention was associated with significant repopulation of the central regions of the scars with cardiomyocytes; this contrasts with some reported data [17]–[18].

### IGF+HGF Supplementation Increases the Long-term Engraftment Rate, but Engrafted Cells Rarely Differentiate into Cardiomyocytes

**Figure 2:**
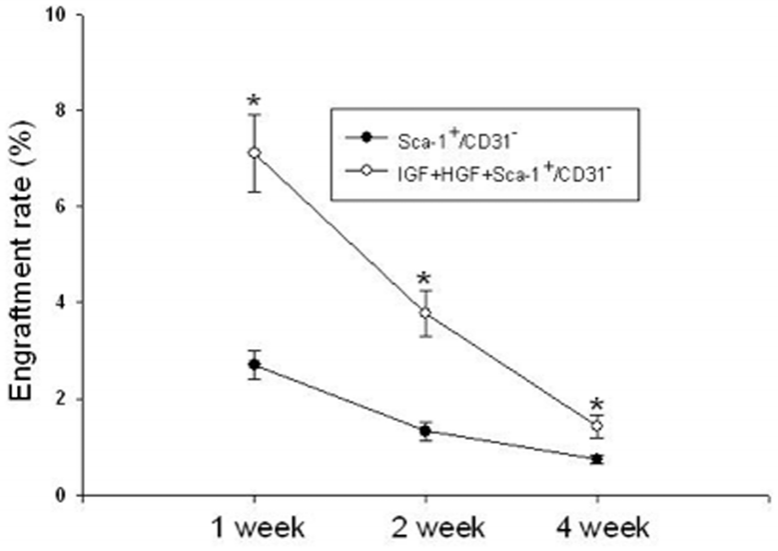
Effects of IGF+HGF supplementation increase the engraftment rates of transplanted Sca-1^+^/CD31^−^ cells. The *y*-axis indicates the number of X-gal and DAPI double stained cells in the whole heart relative to total cell numbers injected. Data were acquired at 1, 2, and 4 weeks after transplantation (n = 6, P<0.01 vs Sca-1^+^/CD31^−^ cells without HGF+IGF). X-gal, 5-bromo-4-chloro-3-indolyl-β-D-galactoside. DAPI, 4, 6-diamidino-2-phenylindole.

The absolute cell engraftment rate was determined by counting the total number of DAPI and ?-galactosidase double positive cells in the entire LV at three post-transplantation time points and then dividing that number by the total number of donor cells injected. IGF+HGF supplementation significantly improved the engraftment rate at all three time points although by 4 weeks post-transplantation, the numbers of engrafted cells were remarkably decreased in both groups (Figure 2).

**Figure 3:**
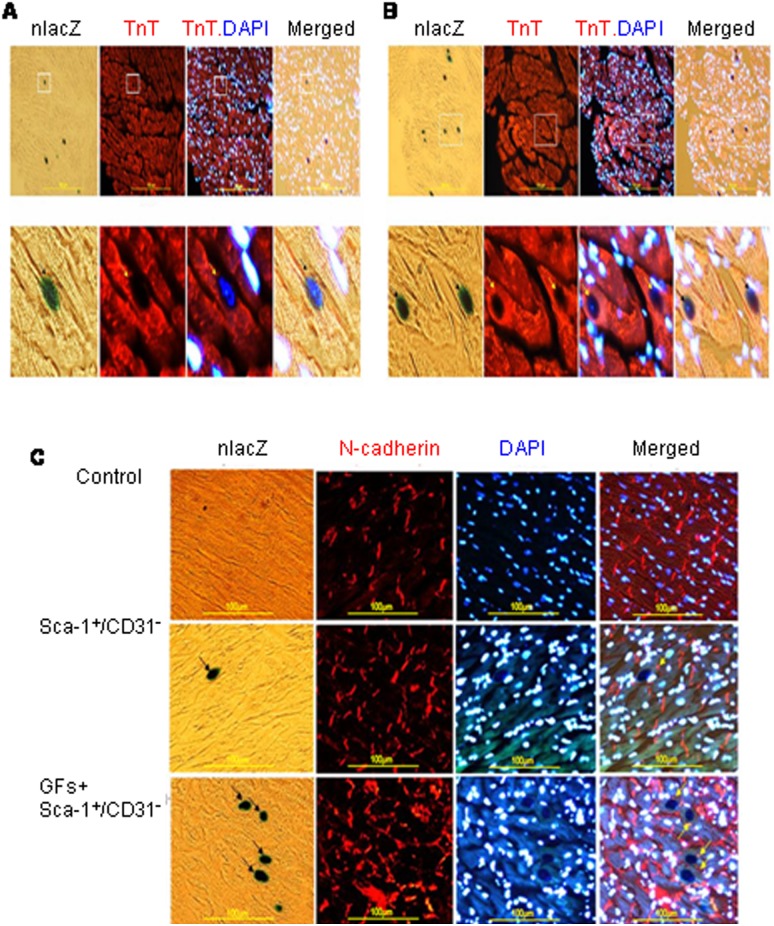
IGF+HGF supplementation increases the number (but not the per cent) of engrafted donor cells expressing cardiogenic markers. (**A**)**.** Representative immunofluorescence staining of engrafted Sca-1^+^/CD31^–^ cells expressing the cardiac-specific marker, troponin T, 2 weeks after transplantation. **Upper panel:** low magnification; **Lower panel:** high magnification; Sequential photomicrographs in each row show nuclear X-gal staining (dark blue), troponin T (red), Troponin and DAPI nuclear staining (light blue), and merged images. Arrow points to triple-positive cells which also showed striations characteristic of cardiomyocytes. (**B**)**.** IGF+HGF supplementation enhanced the number of engrafted Sca-1^+^/CD31^–^ cells expressing cardiomyocyte marker, Troponin T. **Upper panel:** low magnification; **Low panel:** high magnification; Subsequent photomicrographs in each row show nuclear X-gal staining (dark blue), troponin T (red), Troponin and DAPI nuclear staining (light blue), and merged images. Arrows point to triple-positive cells. (**C**)**.** IGF+HGF supplementation enhanced the number of the transplanted Sca-1^+^/CD31^–^ cells expressing gap junction protein, N-cadherin (low magnification). **Upper panel:** tissue from normal heart; **Middle panel:** tissue from Sca-1^+^/CD31^−^ cell transplantation heart; **Low panel:** Tissue from IGF+HGF supplementation heart; Subsequent photomicrographs in each row show nuclear X-gal staining (dark blue), N-cadherin (red), N-cadherin and DAPI nuclear staining (light blue), and merged images. Arrows point to triple-positive cells. DAPI, 4,6-diamidino-2-phenylindol dihydrochloride; X-gal, 5-bromo-4-chloro-3-indolyl-β-D-galactoside.

To determine the fate of engrafted cells, immunofluroscence staining was performed with cardiogenic and endothelial markers. In the Sca-1^+^/CD31^−^ cell transplanted group, about 2% of the engrafted cells (i.e., β-galactosidase and DAPI positive) also stained positive for the cardiogenic marker, troponin T (Figure 3). These triple stained cells were only observed in the non-scar regions of the LV (peri-infarct zone >> remote zone). Among the small number of cells that co-stained positive for β-galactosidase and troponin T, we also identified occasional N-cadherin immunoflurescence staining. The latter observation suggested the presence of some gap junction formation between the differentiated engrafted cells and adjacent cardiomyocytes (Figure 3). Although the IGF+HGF plus Sca-1^+^/CD31^−^ cell group had more engrafted cells that showed evidence of differentiation, this increase was proportional to the increase of engrafted cell numbers in that group. i.e., the likelihood that a given engrafted cell would differentiate was not increased by the co-administration of growth factors. These data, taken together with the fact that engrafted cell numbers were quite low even in the combination therapy group indicated that regeneration of cardiomyocytes from engrafted cells did not account for the structural and functional improvement observed in the Sca-1^+^/CD31^−^ cell treatment groups.

### IGF+HGF Stimulated Endogenous Stem Cells Rarely Differentiate into Cardiomyocytes

**Figure 4:**
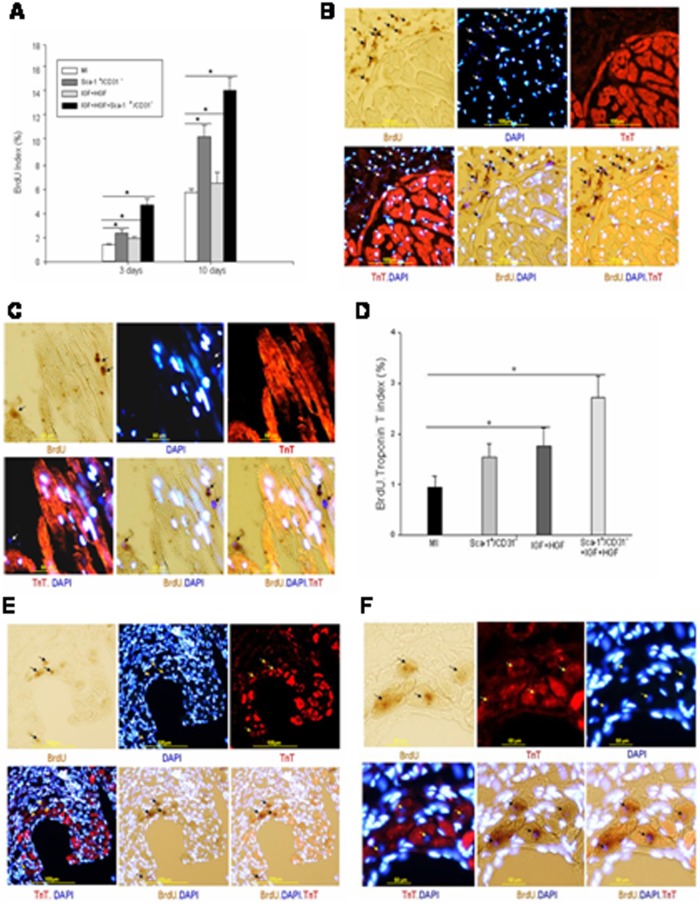
IGF+HGF supplementation enhances cell division of endogenous myocardial cells but not cardiomycyte regeneration from these cells after myocardial injury (MI). (**A**)**.** Bar graph of BrdU index (%) in mouse hearts 3 and 10 days after myocardial injury. (**B, C**)**.** Representative images of BrdU staining positive cells in the infarct zone and interstitial space of per-infarct or remote zone. B: low magnification, C: high magnification. (**D**)**.** Bar graph of frequency of BrdU. Positive cells that co-stained with Troponin T (Tropin T index: %) in the mouse heart 10 days following Mi. (**E,F**)**.** Representative images of BrdU and Troponin T double stained cells in the heart 10 days after myocardial injury. Subsequent photomicrographs in each row show nuclear BrdU staining (Brown), troponin T (red), and DAPI nuclear staining (blue), and merged images. Arrow points to double-positive cells. E: low magnification, F: high magnification.

To determine if cardiomyocytes were regenerated from endogenous stem cells in any group, BrdU *in situ* hybridization and troponin T immunoflurorescence staining were performed. Three days post-acute MI, we observed BrdU positive cells in the hearts from all acute MI groups. Most of these cells were present in the peri-infarct and infarct zones. Sca-1^+^/CD31^−^ cell transplantation alone or IGF+HGF administration without cells increased the number of BrdU positive cells (BrdU index) in the peri-infarct and infarct zones compared to the untreated MI group (MI, 1.40+/−0.08; MI+Sca-1^+^/CD31, 2.44+/−0.21; MI+IGF+HGF, 1.96+/−0.14; n = 6, P<0.05). In contrast, IGF+HGF+Sca-1^+^/CD31 cell administration was associated with two-fold more BrdU positive cells in the peri-infarct zone than present in the aforementioned groups (4.70+/−0.53, n = 6, P<0.01) (Figure 4A). Importantly, we found almost no BrdU and troponin T co-stained cells in any group 3 days after MI. At 10 days post-MI, BrdU indices were further increased in all four groups (MI, 5.78+/−0.27%; MI+IGF+HGF, 6.54+/−0.89%; MI+Sca-1^+^/CD31^−^ cell, 10.42+/−0.95%; MI+IGF+HGF+Sca-1^+^/CD31^−^ cells, 14.25+/1.06%; n = 6, P<0.001 combined treatment vs others). (Figure 4A, B, C). However, once again, we found that only a small number of BrdU positive cells were co-stained with troponin T on day 10 post MI (BrdU Troponin T Index: MI, 0.94+/−0.22%). Sca-1^+^/CD31^−^ cell transplantation slightly increased the number of these cells (Sca-1^+^/CD31^−^ cell, 1.54+/−0.27%, n = 5, P>0.05) compared with MI group. In the IGF+HGF and combined treatment groups this effect was more pronounced at 10 days (IGF+HGF, 1.76+/−0.35%; IGF+HGF+Sca-1^+^/CD31^−^ cells; 2.71+/−0.43%, n = 5, P<0.05 vs other groups) (Figure 4D, E, F). Although these data do suggest that IGF+HGF administration, with or without concomitant cell injection, did stimulate some endogenous stem cells to differentiate into cardiomyocytes; this phenomenon was infrequent and did not likely make a substantial contribution to the therapeutic responses.

### Effects of the Therapeutic Interventions on Peri-infarct Zone Capillary Density

Two weeks after MI, immunofluorescence staining for CD31 antibody demonstrated the presence of more capillaries in the peri-infarct regions of the treated hearts than were present in the untreated MI hearts (Figure S1). The capillary numbers per microscopic field (20x) in the non-IGF+HGF treated groups were as follows: Control, 258.17+/−6.76; MI, 132.33+/−9.45; MI+Sca-1^+^/CD31^−^ cells, 205.83+/−9.19 (n = 6, all P<0.001 vs MI). In the IGF+HGF treated groups, the values were: MI+IGF+HGF, 152+/−9.3 (n = 6, P<0.05 vs MI); MI+IGF+HGF+Sca-1^+^/CD31^−^, 260.1+/−7.39 (n = 6, P<0.001 vs the all but the non-MI Controls) (Figure S1). However, LacZ positive cells expressing CD31 were rarely present in any of the cell treated groups. The latter data indicate that differentiation or fusion based generation of CD31^+^ cells from engrafted cells was minimal.

### IGF+HGF Treatment Protects Stressed Sca-1^+^/CD31^−^ cells *In vitro*

**Figure 5:**
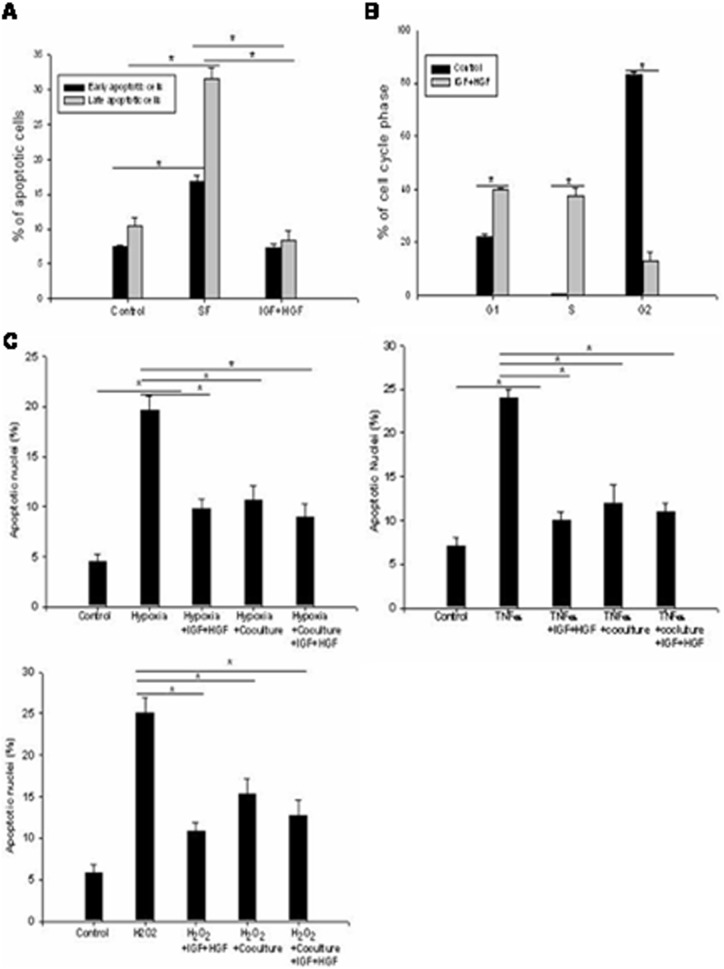
*In vitro* data showing that IGF+HGF stimulation enhances Sca-1^+^/CD31^−^ cell survival and proliferation and protects co-cultured stressed HL-1 from apoptosis. (**A**)**.** FACS analysis showed that IGF+HGF treatment significantly decreased serum free medium induced Sca-1^+^/CD31^−^ cell apoptosis (n = 6, P<0.01). Apoptotic cells were determined using FITC Annexin V staining. (**B**)**.** FACS analysis demonstrated that IGF+HGF treatment stimulated Sca-1^+^/CD31^−^ cells to enter the cell cycles as indicated by more cells entering G1 and S phases (n = 6, P<0.01). (**C**)**.** Addition of IGF+HGF to HL-1 cells or co-culture of the cells with Sca-1^+^/CD31^−^ cells inhibited stressor (hypoxia, H_2_O_2_ and TNFα) induced HL-1 cell apoptosis (n = 6, P<0.001). Data are presented as mean+/−SEM.

It is well known that IGF and HGF participate in the regulation of cell survival and proliferation. To determine why IGF+HGF co-administration increased the rate of cell engraftment, we asked if IGF+HGF treatment might significantly inhibit apoptosis in Sca-1^+^/CD31^−^ cells exposed to a known stressor *in vitro.* Flow cytometry data analysis showed that survival of Sca-1^+^/CD31^−^ cells exposed to serum free medium for 48 hours was significantly increased by treatment with IGF+HGF. In IGF+HGF treated stressed cells, Annexin positive cell populations including early (FITC staining positive) and late (FITC and PI double staining positive) evidence of apoptosis remained at control levels (i.e., the levels present in unstressed cells) as compared to the marked elevations present in stressed cells not treated with growth factors. Stressed cells showing early evidence of early apoptosis were less frequent in treated group (7.36+/−0.47%) than in the untreated group (16.77+/−0.91%) (n = 6, p<0.001)]. Similarly, stressed cells showing late evidence of apoptosis were less frequent in the treated (8.42+/−1.25%) than the untreated group (31.65+/−1.52%; (n = 6, P<0.001). (Figure 5A, Figure S2). We also found that IGF+HGF treatment caused more cells to enter the G1 and S phases of the cell cycle in the treated group (Figure 5B, Figure S3).

*In vitro* experiments were also employed to test the hypothesis that paracrine mediated suppression of apoptosis of native cardiomyocytes post-acute MI in the *in vivo* heart might occur as a consequence of exposure to Sca-1^+^/CD31^−^ cells. Therefore, Sca-1^+^/CD31^−^ cells were co-cultured with mouse atrium derived HL-1 cells with or without IGF+HGF supplementation. The number of apoptotic nuclei of HL-1 cells was assessed by H33342 staining following exposure to different stressors (hypoxia, H_2_O_2_ or TNF??. We found that both Sca-1^+^/CD31^−^ cell co-culture and IGF+HGF supplementation decreased HL-1 cell apoptosis in all stressor groups (Figure 5C). Moreover, combined cell and growth factor co-culture was not more effective than observed with the single interventions (data not shown). Hence, synergy between the two interventions was absent at the concentrations of Sca-1^+^/CD31^−^ cells and growth factors used in the co-culture studies.

### IGF+HGF Decreases Apoptosis of Native Cardiomyocytes in the Post-MI Heart

**Figure 6:**
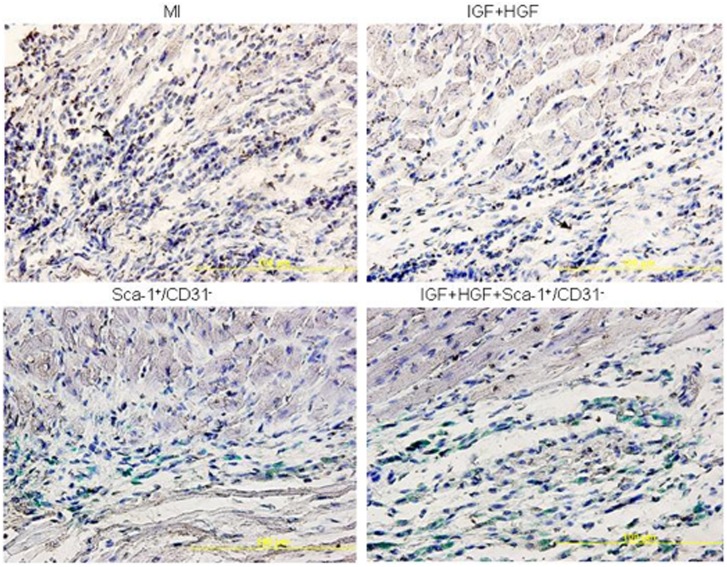
IGF+HGF administration is associated with decreased apoptosis (TUNEL staining) in myocardium 3 days after MI.

Consistent with the *in vitro* data, TUNEL staining of heart tissues demonstrated Sca-1^+^/CD31^−^ cell transplantation alone decreased the apoptotic indices in the peri-infarct zones 3 days post-acute MI (MI, 25.62+/−1.56%; MI+Sca-1^+^/CD31^−^ cell, 11.64+/−1.21%; n = 6, P<0.001). IGF+HGF+Sca-1^+^/CD31^−^ cell treatment enhanced the anti-apoptotic effect of Sca-1^+^/CD31^−^ cell transplantation further (6.67+/−1.01%, n = 6, P<0.05 vs Sca-1^+^/CD31^−^ alone) (Figure 6). Additionally, injection of IGF+HGF alone decreased the apoptotic index 3 days after MI (14.86+/−3.24%; n = 6, P<0.05 compared to MI). These data indicate that Sca-1^+^/CD31^−^cell transplantation ± IGF+HGF injection is protective with regard to *in vivo* cardiomyocyte apoptosis but, unlike the case with the *in vitro* data, the combined intervention was more effective than single interventions in the *in vivo* heart.

### mRNAs Profiling of Sca-1^+^/CD31^−^ and IGF+HGF Treated Sca-1^+^/CD31^−^ Cells

**Figure 7:**
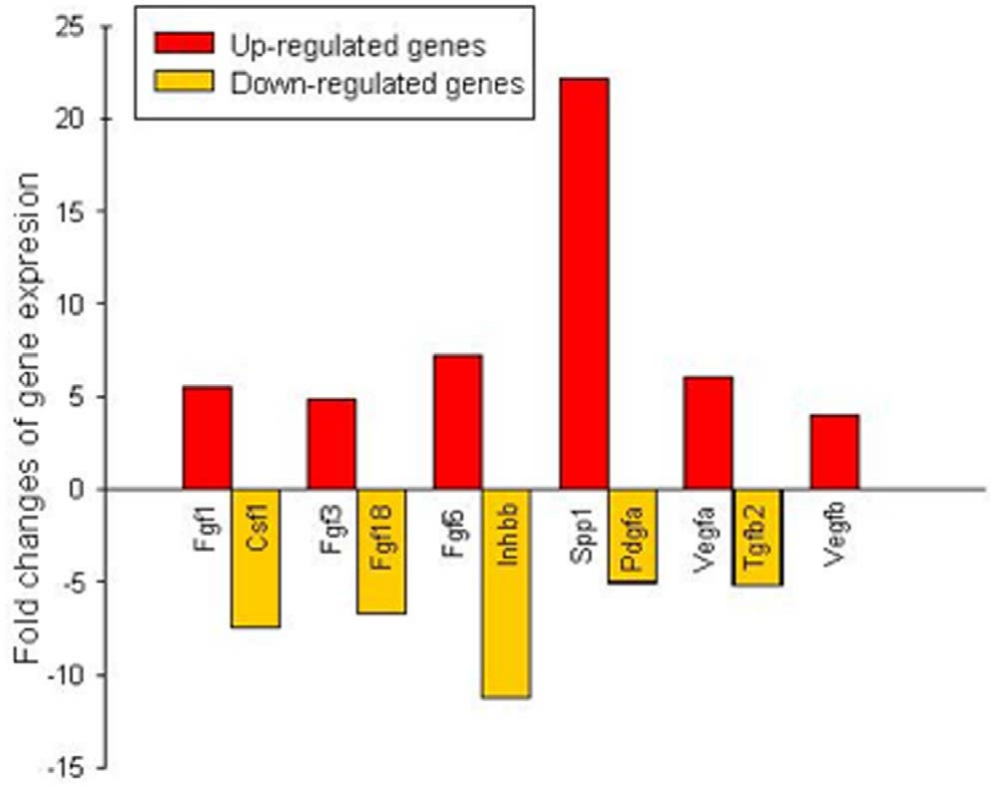
Supperarray profiling of 88 growth factor related genes in Sca1^+^/CD31^−^ cell and IGF+HGF treated Sca-1^+^/CD31^−^ cells. The fold differences plotted for each gene are calculated from via the ΔΔC_t_ method and raw data normalized to a panel of housekeeping gene.

Understanding the effects of IGF+HGF on gene expression patterns in Sca-1^+^/CD31^−^ cells is crucial to understanding the *in vivo* effects of combination therapy. Therefore, using growth factor associated gene superarray analyses, we profiled both Sca-1^+^/CD31^−^cells and IGF+HGF treated Sca-1^+^/CD31^−^ cells. The Sca-1^+^/CD31^−^ cell mRNA expression patterns are shown in Table 1; in this Table the growth factor associated mRNAs that are highly expressed (defined as Ct value greater than 25) are included. IGF+HGF treatment significantly altered the expression patterns of a number of growth factor related mRNAs (Table 1, Figure 7 and Figure S4). 6 mRNAs were up-regulated and 5 mRNAs were down-regulated following exposure of the cells to IGF+HGF. These data suggest that one effect of exposure of Sca-1^+^/CD31^−^ cells to IGF+HGF was increased expression of mRNAs involved in the synthesis of growth factors and cell cycle regulators. A well-known effect of ligand stimulation of growth factor receptors is activation of Akt and its downstream signaling pathways. We speculate that, as was shown to be the case for marrow derived mesenchymal stem cells overexpressing Akt, that the growth and trophic factor secretion by IGF+HGF treated Sca-1^+^/CD31^−^ cells was also significantly increased [10], [30]–[32]. More detailed investigation of this issue is warranted.

**Table 1:** High expression of growth factor associated genes in mouse heart derived Sca-1^+^/CD31^−^ cells.

Gene	Expression	
Well	Description	Gname
A04	Bone morphogenetic protein 1	Bmp1
B02	Colony stimulating factor 1 (macrophage)	Csf1
B06	Chemokine (C-X-C motif) ligand 12	Cxcl12
B11	Fibroblast growth factor 11	Fgf11
D02	C-fos induced growth factor	Figf
D04	Growth differentiation factor 11	Gdf11
D09	Insulin-like growth factor 1	Igf1
E09	Inhibin alpha	Inha
E11	Inhibin beta-B	Inhbb
E12	Kit ligand	Kit ligand
F01	Left right determination factor 1	Lefty1
F06	Nerve growth factor	Ngf
F10	Platelet derived growth factor, alpha	Pdgfa
F12	Rabaptin, RAB GTPase binding effector protein 1	Rabep1
G01	S100 calcium binding protein A6 (calcyclin)	S100a6
G02	Secreted phosphoprotein1	Spp1
G06	Transforming growth factor, beta 1	Tgfb1
G07	Transforming growth factor, beta 2	Tgfb2
G09	Vascular endothelial growth factor A	Vegfa
G10	Vascular endothelial growth factor B	Vegfb
G11	Vascular growth factor C	Vegfc
G12	Zinc finger protein 91	Zfp91

## Discussion

We have reported that intra-myocardial injection of mouse heart derived Sca-1^+^/CD31^−^ cells at the time of induction of myocardial injury attenuates the adverse structural remodeling and functional declines consequent to the injury [26]. We also found, using Sca-1 (heterozygous) knockout mice, that the severity of post-acute MI LV remodeling was greater than that present in wild type controls [27]. Taken together, these observations suggested that both exogenous and endogenous myocardial Sca-1^+^/CD31^−^ cells somehow contributed to the repair processes that occur post-acute MI.

The present study explores the mechanisms underlying the beneficial effects of transplantation of heart derived Sca-1^+^/CD31^−^ cells and asks whether these effects can be amplified by the co-administration of growth factors (IGF and HGF) at the time of cell transplantation. The major findings are: 1. The beneficial effects of Sca-1^+^/CD31^−^ cell transplantation in the context of acute MI are confirmed; 2. Sca-1^+^/CD31^−^ cells exert anti-apoptotic effects on stressed cardiomyocytes both under *in vitro* and *in vivo* conditions; 3. Sca-1^+^/CD31^−^ cell transplantation attenuates the decrease of capillary density that usually is present in the peri-infarction zone of post-MI hearts; 4. All of these responses are significantly enhanced in hearts in which IGF and HGF were co-administered with cells; 5. Differentiation of endogenous stem cells into cardiomyocytes, although enhanced by all interventions was still minimal in all groups; 6. Sca-1^+^/CD31^−^ cell engraftment was enhanced by co-administration of growth factors, but differentiation of engrafted cells into cardiomyocytes or vascular cells was infrequent; and 7. The *in vitro* exposure of Sca-1^+^/CD31^−^ cells to IGF+HGF was associated with significant up-regulation or down-regulation of the expression of mRNAs that are likely linked to trophic factor generation/secretion by these cells.

Collectively, these data support previous reports that indicate that the protective properties of transplanted stem cells can be mediated via paracrine mechanisms [10], [30]–[32]. Somewhat surprisingly, in contrast to some previously published work, cardiomyocyte regeneration from either transplanted or endogenous stem cells did not appear to play a major role in the therapeutic response [17]–[18], [25]. Our data are also compatible with the prevailing view that induction of vasculogenesis (and/or preservation of native capillaries) is commonly associated with cell transplantation (and/or growth factor administration) and that increased capillary density contributes to the observed myocardial protection.

### IGF+HGF Added to Cell Transplantation Increases the Rates of Cell Engraftment and Native Cardiomyocyte Survival

It is well established that post-reperfusion oxidative stress, inflammatory processes, and other adverse events that occur in the injured region of the acutely infarcted heart contribute to the occurrence of ischemic (necrotic) cellular death as well as to early and late apoptosis of cardiomyocytes [33]. The adverse characteristics of the tissue micro-environment are also presumed to negatively affect the number of cells that persistently engraft following direct myocardial transplantation early during the course of an MI.

The administration of IGF+HGF at the time of cell transplantation essentially doubled the number of engrafted cells present at all time points examined. Hence, as previously reported, trophic factor co-administration or genetic manipulation of donor cells to enhance their rate of secretion of trophic factors enhances both engraftment rates and therapeutic effects [10], [30]–[32]. However, despite the increased engraftment rate present in hearts also receiving IGF+HGF, the late engraftment rates were low in all groups. Moreover, the differentiation of engrafted cells or endogenous stem cells into cardiomyocytes and vascular cells was not frequent. It seems reasonable to conclude from these data that the benefits of cell transplantaton ± IGF+HGF were due to paracrine mediated protection of native cardiomyocytes (and perhaps vascular cells).

In humans, native cardiomyocyte apoptosis that occurs early after acute MI is present mainly in the infarct and peri-infarct border zones [34]. In the present study we found that all treatments significantly inhibited the early phase of native cardiomyocyte apoptosis (i.e., within 3 days post-MI) and that these effects were most prominent in the combined treatment group. This early anti-apoptotic effect was also observed in our *in vitro* studies. For example, administration of IGF+HGF decreased the severity of apoptosis present in Sca-1^+^/CD31^−^ cells exposed to serum deprivation and co-culture of Sca-1^+^/CD31^−^ cells with atrium derived HL-1 cells significantly inhibited apoptosis of these cells when they were challenged with stressors such as hypoxia, TNFα or H_2_O_2_.

Of interest, HL-1 cell co-culture with Sca-1^+^/CD31^−^ cells alone, culture with IGF+HGF alone and, the culture with cells plus IGF+HGF all yielded comparable results. The latter findings suggest that, in the *in vitro* studies, the ratio of Sca-1^+^/CD31^−^ to HL-1 cells was high enough so that the effects of the presumed secreted trophic factors were already optimal. Hence, additional stimulation of the secretory output Sca-1^+^/CD31^−^cells and/or the direct effects of the IGF+HGF on the HL-1 cells could not elicit additional protection. In this regard, in our *in vivo* studies, the ratio of engrafted cells to native cardiomyocytes was much lower than that presents in the *in vitro* HL-1 cell studies and likely could not elicit maximal protective effects. Hence, under *in vivo* conditions, it is possible that the addition of IGF+HGF was able to increase therapeutic effectiveness of cell transplantation by enhancing trophic factor secretion by engrafted cells (as well as by direct effects of IGF+HGF on injured cardiomyocytes).

Our *in vivo* and *in vitro* data are consistent with published reports [10], [30]–[32] that showed that the early anti-apoptotic effects of cell therapy occurred in less than 3 days, preceded the later increase in myocardial capillary density. These authors also showed that trophic factors were released by their transplant cells and that over-expression of Akt in these cells was associated with a marked increase in their rates of secretion of trophic factors, and, thereby, of their therapeutic effectiveness [10].

### Effects of Interventions on Infarct Border Zone Capillary Density

All therapeutic interventions significantly attenuated the reduction in capillary density that occurred in the infarct border zone of untreated hearts. In fact, the combination of cell and trophic factor administration completely prevented this fall in capillary density. Of interest, the response to trophic factor injection alone tended to be smaller than that to cell therapy alone. Because, there was only a small direct contribution of engrafted or endogenous stem cells (via differentiation) to the increased capillary numbers, we conclude that the increase of capillary density can also be attributed to paracrine mechanisms acting on endogenous vascular cell precursors.

### The Low Rate of Generation of New Cardiomyocytes Present in this Acute MI Model is Comparable to that Reported in Many, but not All Earlier studies

Some published reports have indicated that IGF+HGF administration (alone) to newly infarcted rodent and dog hearts did result in significant cardiomyocyte generation from endogenous CSCs [17]–[18], [25]. However, even in the combined treatment group, there was evidence of only modest cardiomyocyte regeneration from either transplanted or endogenous stem cells. We have no obvious explanation for why our results differ from some previously reported results.

## Summary

The major effects of the Sca-1^+^/CD31^−^ cell transplantation (with or without added IGF+HGF) are paracrine in nature. The data supporting this view include: 1. decreased rates of apoptosis of injured cardiomyocytes, 2. preservation of capillary density in the peri-infarct zone of the LV which improved perfusion capacity and presumably cardiomyocyte contractile function, and 3. possible enhancement of suggested beneficial interactions between the vasculature and cardiomyocytes [35]. It is possible that IGF+HGF administration might be a useful adjuvant to therapeutic cell transplantation at the time of acute revascularization therapy for acute MI. Moreover, it may be that the co-administration of IGF+HGF with Sca-1^+^/CD31^−^ cells during a later phase of MI recovery might be associated with more significant cardiomyocyte regeneration [6], [36].

## Supporting Information

Figure S1 **IGF+HGF added to cell transplantation results in increased vascular density as compared to cell transplantation alone.** **(A).** Immunoflurorescence staining for CD31 (green) and DAPI in peri-infarction zone 2 weeks post-MI in untreated, Sca-1^+^/CD31^–^ cell, IGF+HGF and Sca-1^+^/CD31^–^ cell, and IGF+HGF treated hearts increases density and this response was greatest in the combination treatment group **(B).** Mean number of CD31 stained capillaries in peri-infarct regions of the experimental groups (p<0.05 for indicated comparisons. DAPI, 4,6-diamidino-2-phenylindol dihydrochloride; MI, myocardial infarction; CD31, PECAM-1. Data are presented as mean+/−SEM.
(TIF)

Figure S2 **The representative images of Sca-1^+^/CD31^−^ cells stained with FITC Annexin V and propidium iodide (PI).** **(A).** Selected Sca-1^+^/CD31^−^ cell population. **(B).** Dot plot of Sca-1^+^/CD31^−^ cells stained with PI only. **(C).** Dot plot of Sca-1^+^/CD31^−^ cells stained with FITC Annexin V only. **(D).** Dot plot of Sca-1^+^/CD31^−^ cell stained with FITC-Annexin V and PI.
(TIF)

Figure S3 **The representative images of cell cycle analysis with flow cytometry.** **(A).** Sca-1^+^/CD31^−^ cells cultured in basal medium+0.5% FBS for 24 hours after 12 hours synchronization. **(B).** Sca-1^+^/CD31^−^ cell treated with IGF+HGF in basal medium+0.5% FBS for 24 hours.
(TIF)

Figure S4 **Schachard plot shows up-regulated and down-regulated genes after Sca-1^+^/CD31^−^cell treated with IGF+HGF.** Supperarray profiling of 88 growth factor related genes of Sca-1^+^/CD31^−^ cell and IGF+HGF treated Sca-1^+^/CD31^−^ cells. The data is described as having a 4 fold increase or decrease as a scale for determining up-regulation or down-regulation.
(TIF)

Text S1 **Sca-1^+^/CD31^–^ and Sca-1^−^/CD31^−^ cell isolation and fluorescence-activated cell sorting analysis.**
(DOC)

Text S2 **Animal surgery and cell transplantation.**
(DOC)

Text S3 **Echocardiography.**
(DOC)

Text S4 **Cell engraftment rates and differentiation status.**
(DOC)

References S1 (DOCX)
