# Supplementary figures and images for: Intra-Myocardial Injection of Both Growth Factors and Heart Derived Sca-1+/CD31− Cells Attenuates Post-MI LV Remodeling More Than Does Cell Transplantation Alone: Neither Intervention Enhances Functionally Significant Cardiomyocyte Regeneration
Source: PLoS One. 2014 Jun 11;9(6):e95247. doi: 10.1371/journal.pone.0095247 (PMC4053321; doi:10.1371/journal.pone.0095247)

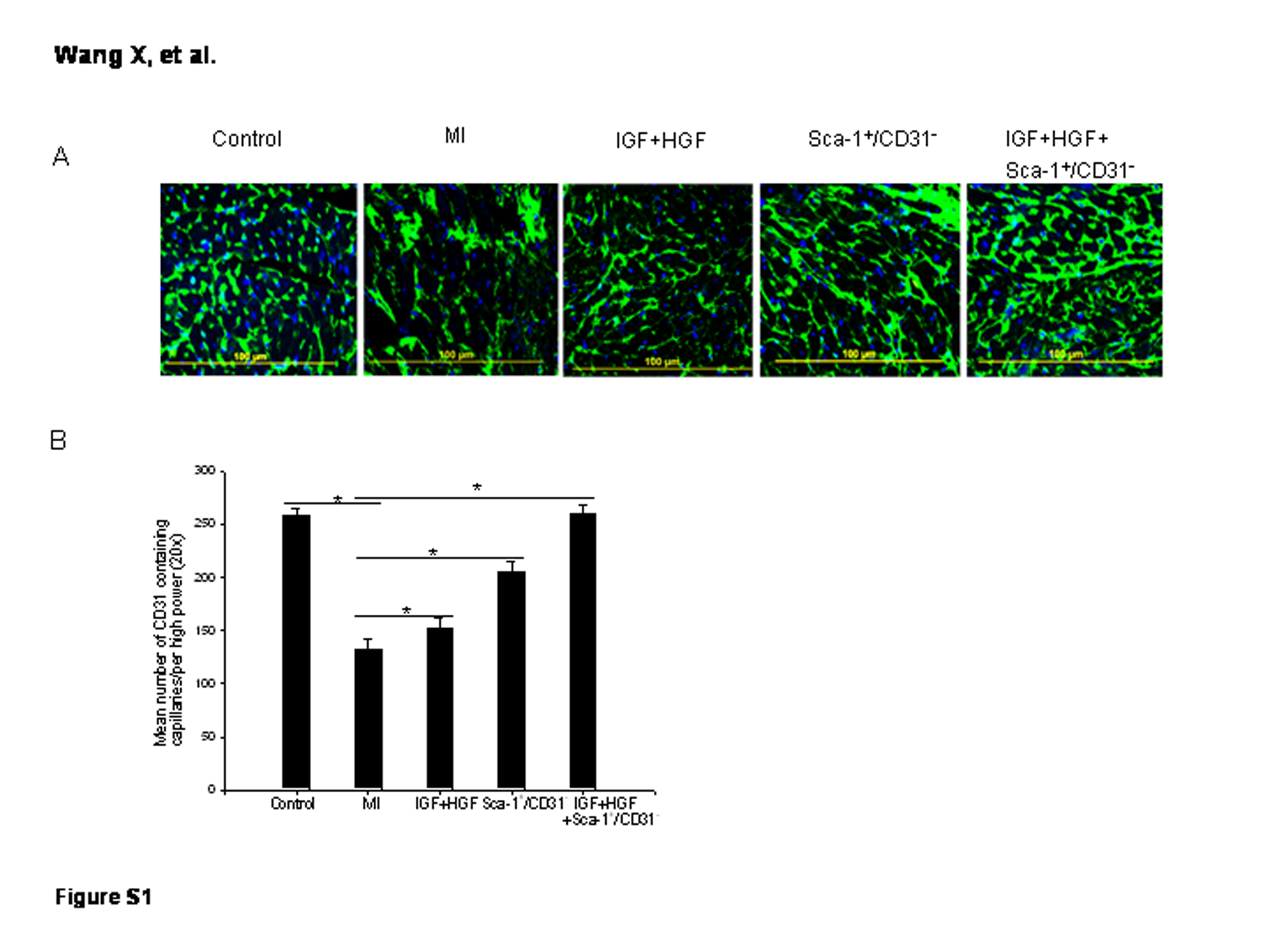

Supplement: Figure S1 — IGF+HGF added to cell transplantation results in increased vascular density as compared to cell transplantation alone. (A). Immunoflurorescence staining for CD31 (green) and DAPI in peri-infarction zone 2 weeks post-MI in untreated, Sca-1+/CD31– cell, IGF+HGF and Sca-1+/CD31– cell, and IGF+HGF treated hearts increases density and this response was greatest in the combination treatment group (B). Mean number of CD31 stained capillaries in peri-infarct regions of the experimental groups (p<0.05 for indicated comparisons. DAPI, 4,6-diamidino-2-phenylindol dihydrochloride; MI, myocardial infarction; CD31, PECAM-1. Data are presented as mean+/−SEM. (TIF) [file pone.0095247.s001.tif]

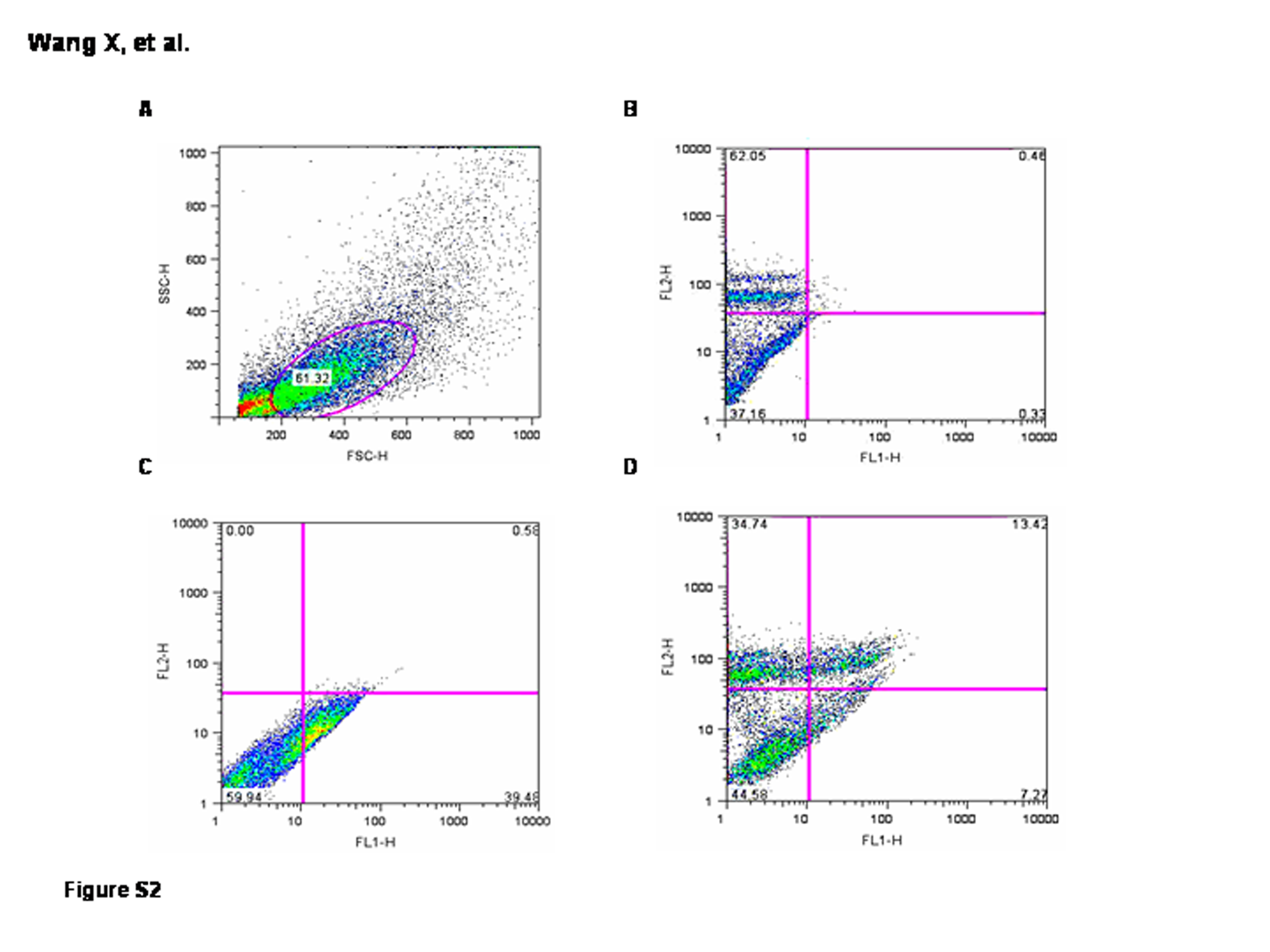

Supplement: Figure S2 — The representative images of Sca-1+/CD31− cells stained with FITC Annexin V and propidium iodide (PI). (A). Selected Sca-1+/CD31− cell population. (B). Dot plot of Sca-1+/CD31− cells stained with PI only. (C). Dot plot of Sca-1+/CD31− cells stained with FITC Annexin V only. (D). Dot plot of Sca-1+/CD31− cell stained with FITC-Annexin V and PI. (TIF) [file pone.0095247.s002.tif]

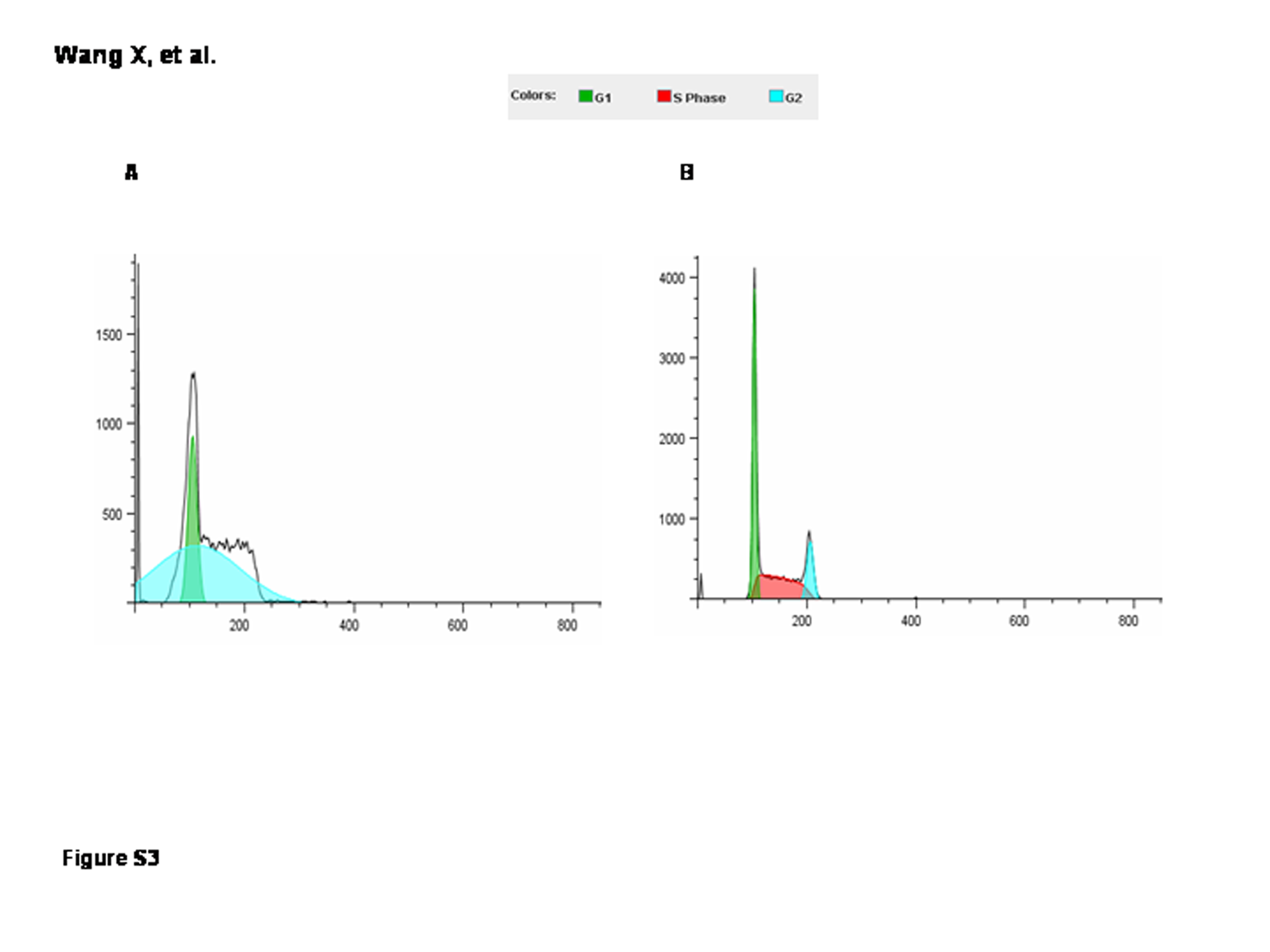

Supplement: Figure S3 — The representative images of cell cycle analysis with flow cytometry. (A). Sca-1+/CD31− cells cultured in basal medium+0.5% FBS for 24 hours after 12 hours synchronization. (B). Sca-1+/CD31− cell treated with IGF+HGF in basal medium+0.5% FBS for 24 hours. (TIF) [file pone.0095247.s003.tif]

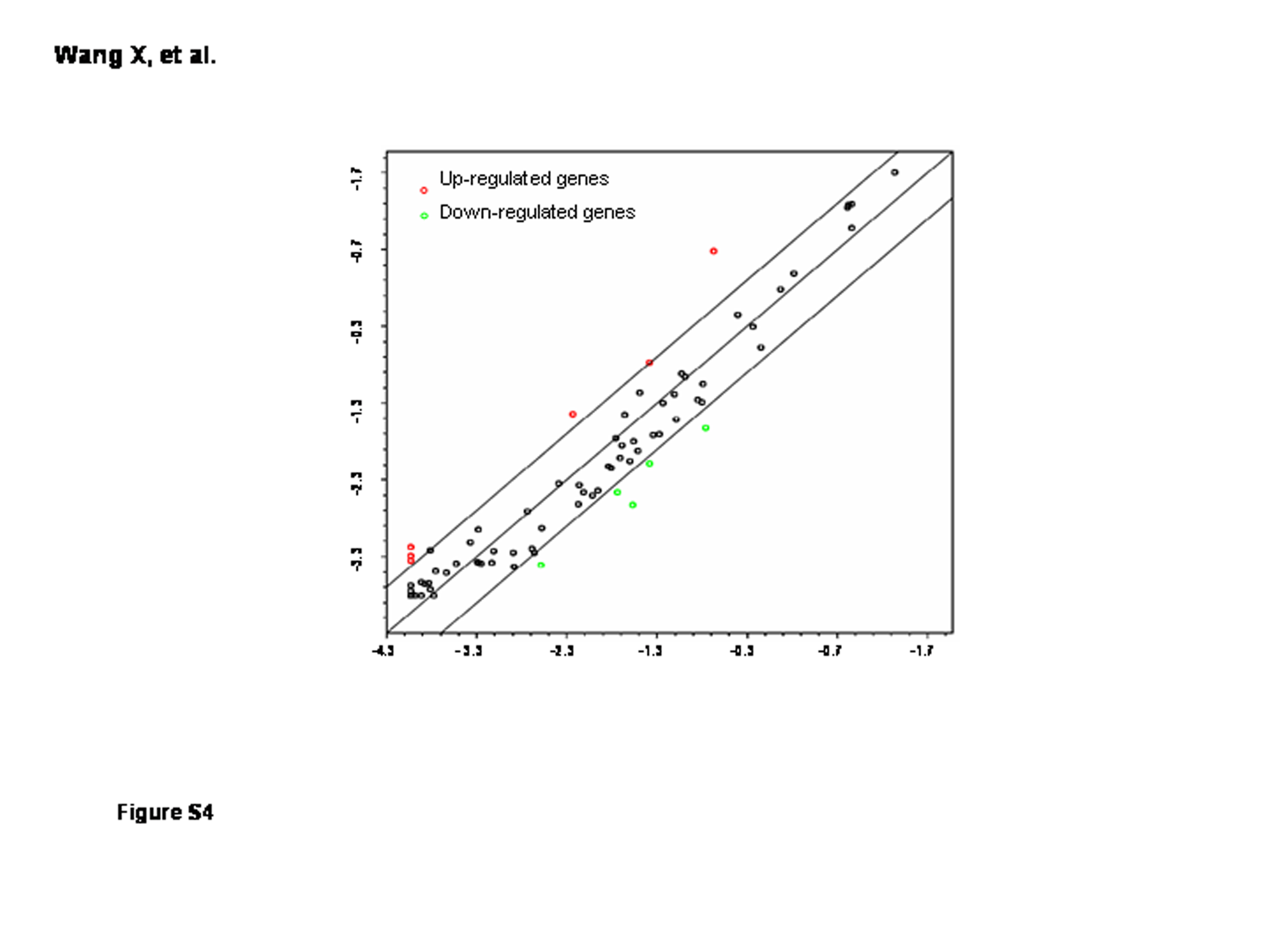

Supplement: Figure S4 — Schachard plot shows up-regulated and down-regulated genes after Sca-1+/CD31−cell treated with IGF+HGF. Supperarray profiling of 88 growth factor related genes of Sca-1+/CD31− cell and IGF+HGF treated Sca-1+/CD31− cells. The data is described as having a 4 fold increase or decrease as a scale for determining up-regulation or down-regulation. (TIF) [file pone.0095247.s004.tif]
