# Supplementary material for: Intra-Myocardial Injection of Both Growth Factors and Heart Derived Sca-1+/CD31− Cells Attenuates Post-MI LV Remodeling More Than Does Cell Transplantation Alone: Neither Intervention Enhances Functionally Significant Cardiomyocyte Regeneration
Source: PLoS One. 2014 Jun 11;9(6):e95247. doi: 10.1371/journal.pone.0095247 (PMC4053321; doi:10.1371/journal.pone.0095247)
Supplement: Text S1 — Sca-1+/CD31– and Sca-1−/CD31− cell isolation and fluorescence-activated cell sorting analysis. (DOC) [file pone.0095247.s005.doc]

**Text S1. Sca-1^+^/CD31^–^ and** Sca-1^-^/CD31^-^ **cell isolation and fluorescence-activated cell sorting analysis.**

Briefly, the mouse heart was perfused with cold Ca^2+^-Mg^2+^-free phosphate-buffered solution (PBS) (Invitrogen), and digested two times for 5 minutes at 37^o^C with 0.2% trypsin (Invitrogen) and 0.1% collagenase IV (Worthington Biochemical Corp). The dissociated cells were discarded and the remaining tissues washed with Iscover’s Modified Dulbecco’s Medium (IMDM) supplemented with 10% fetal calf serum, 100U/ml penicillin G, 100µg/ml streptomycin and 2mmol L-glutamine were cultured as explants in IMDM medium at 37^o^C and 5% CO_2_. After 2 weeks, a layer of fibroblast-like cells was generated from the explants and expanded. Sca-1^+^/CD31^-^ and Sca-1^-^/CD31^-^ cells were isolated from the fibroblast-like cells using a magnetic sorting system and expanded in cardiac sphere growth medium (CGM) containing DMEM/F12, 10% FBS, 200 mmol/L L-glutamine, 0.1mmol/L β-mercaptoethanol, 1% nonessential amino acids, 1000 units/ml leukemia inhibitory factor, 0.1unit/ml thrombin and 5ng/ml basic fibroblast growth factor as previously described [1,2,4] (in File S1).
