# Supplementary material for: Intra-Myocardial Injection of Both Growth Factors and Heart Derived Sca-1+/CD31− Cells Attenuates Post-MI LV Remodeling More Than Does Cell Transplantation Alone: Neither Intervention Enhances Functionally Significant Cardiomyocyte Regeneration
Source: PLoS One. 2014 Jun 11;9(6):e95247. doi: 10.1371/journal.pone.0095247 (PMC4053321; doi:10.1371/journal.pone.0095247)
Supplement: Text S2 — Animal surgery and cell transplantation. (DOC) [file pone.0095247.s006.doc]

**Text S2. Animal surgery and cell transplantation.**

Mice were anesthetized by intraperitoneal injections of sodium pentobarbital (35 mg/kg) and lidocaine hydrochloride (10 mg/kg), instrumented with a standard limb lead II electrocardiogram (ECG), intubated, and mechanically ventilated using a small-animal respirator (Harvard Apparatus). Under a stereomicroscope, the heart was exposed via left thoracotomy and the left anterior coronary artery (LAD) was ligated with a 9-0 surgical suture to produce a myocardial infarction. Intramyocardial injections of saline, IGF+HGF (each at a concentration of 100ng/ml in saline; 5-7 ng/heart of each growth factor administered per heart), heart-derived Sca-1^+^/CD31^–^ cells in saline (± growth factors), and heart derived Sca-1^-^/CD31^-^ cells were injected at five sites in the peri-infarct zone immediately following LAD ligation (total injected volume of 50-70 µl/heart; total number of cells injected = 1 x 10^6^/heart).
