# Supplementary material for: Intra-Myocardial Injection of Both Growth Factors and Heart Derived Sca-1+/CD31− Cells Attenuates Post-MI LV Remodeling More Than Does Cell Transplantation Alone: Neither Intervention Enhances Functionally Significant Cardiomyocyte Regeneration
Source: PLoS One. 2014 Jun 11;9(6):e95247. doi: 10.1371/journal.pone.0095247 (PMC4053321; doi:10.1371/journal.pone.0095247)
Supplement: Text S3 — Echocardiography. (DOC) [file pone.0095247.s007.doc]

**Text S3. Echocardiography.**

Mice were lightly anesthetized using ketamine HCl (25 mg/kg i.p.) and xylazine (10 mg/kg i.p.), and chest fur was removed using a depilatory cream. Images were obtained in left- and right-parasternal long- and short-axis views for measurements of left ventricular (LV) volumes in diastole and systole, LV internal diameters in diastole and systole, as well as LV posterior wall and interventricular septal thicknesses in diastole. LV ejection fraction (LVEF) was calculated for each animal. At the end of the experiment, the left ventricles were excised. Infarct size was calculated and expressed as a percentage of LV surface area (NIH Image J program, http://rsb.info.nih.gov/ij)[4,5] (in File S1).
