# Supplementary material for: Intra-Myocardial Injection of Both Growth Factors and Heart Derived Sca-1+/CD31− Cells Attenuates Post-MI LV Remodeling More Than Does Cell Transplantation Alone: Neither Intervention Enhances Functionally Significant Cardiomyocyte Regeneration
Source: PLoS One. 2014 Jun 11;9(6):e95247. doi: 10.1371/journal.pone.0095247 (PMC4053321; doi:10.1371/journal.pone.0095247)
Supplement: Text S4 — Cell engraftment rates and differentiation status. (DOC) [file pone.0095247.s008.doc]

**Text S4. Cell engraftment rates and differentiation status.**

Mouse hearts that had received LacZ transduced Sca-1^+^/CD31^–^ cells were fixed with 2% paraformaldehyde and subjected to β-galactosidase staining. Hearts were photographed, embedded into Tissue-Tek OCT (Fisher Scientific), transversely sectioned into 8-µm slices using a cryostat, and stained with troponin T (Labvision), and N-cadherin (Novus Biologicals) antibodies as previously described. The sections were visualized using fluorescence-labeled secondary antibodies (Molecular Probes Inc., Eugene, OR, http://probes.invitrogen.com). The total number of cell nuclei per high power (20x) was identified by DAPI (Sigma-Aldrich) staining. The engraftment rate was determined by counting DAPI and 5-bromo-4-chloro-3-indolyl-β-D-galactoside (X-gal) double-stained positive nuclei in the whole heart and dividing that number by 1 million donor cells and multiplying by 100%.
