# Supplementary material for: Intra-Myocardial Injection of Both Growth Factors and Heart Derived Sca-1+/CD31− Cells Attenuates Post-MI LV Remodeling More Than Does Cell Transplantation Alone: Neither Intervention Enhances Functionally Significant Cardiomyocyte Regeneration
Source: PLoS One. 2014 Jun 11;9(6):e95247. doi: 10.1371/journal.pone.0095247 (PMC4053321; doi:10.1371/journal.pone.0095247)
Supplement: References S1 — (DOCX) [file pone.0095247.s009.docx]

**REFERENCES S1.**

1. Messina E, De Angelis L, Frati G, Morrone S, Chimenti S, et al (2004). Isolation and expansion of adult cardiac stem cells from human and murine heart. Circ Res 95:911-921.

2. Smith RR, Barile L, Cho HC, Leppo MK, Hare JM, et al (2007). Regenerative potential of cardiosphere-derived cells expanded from percutaneous endomyocardial biopsy specimens. Circulation 115:896-908.

3. Tolar J, Wang X, Braunlin E, McElmurry RT, Nakamura Y, et al (2007). The host immune response is essential for the beneficial effect of adult stem cells after myocardial ischemia. Exp Hematol 35:682-690.

4. Tang YL, Shen L, Qian K, Phillips MI (2007)**.** A novel two-step procedure to expand cardiac Sca-1+ cells clonally. Biochem Biophys Res Commun 359:877-883.

**5.** Wang X, Hu Q, Nakamura Y, Lee J, Zhang G, et al (2006). The role of the Sca-1+/CD31- cardiac progenitor cell population in postinfarction left ventricular remodeling. Stem Cells 24:1779-1788.
